# Fungal communities in ancient peatlands developed from different periods in the Sanjiang Plain, China

**DOI:** 10.1371/journal.pone.0187575

**Published:** 2017-12-13

**Authors:** Zhenqing Zhang, Xue Zhou, Lei Tian, Lina Ma, Shasha Luo, Jianfeng Zhang, Xiujun Li, Chunjie Tian

**Affiliations:** 1 Key Laboratory of Mollisols Agroecology, Northeast Institute of Geography and Agroecology, Chinese Academy of Sciences, Changchun, China; 2 Key Laboratory of Wetland Ecology and Environment, Northeast Institute of Geography and Agroecology, Chinese Academy of Sciences, Changchun, China; 3 University of the Chinese Academy of Sciences, Beijing, China; Friedrich Schiller University, GERMANY

## Abstract

Peatlands in the Sanjiang Plain could be more vulnerable to global warming because they are located at the southernmost boundary of northern peatlands. Unlike bacteria, fungi are often overlooked, even though they play important roles in substance circulation in the peatland ecosystems. Accordingly, it is imperative that we deepen our understanding of fungal community structure and diversity in the peatlands. In this study, high-throughput Illumina sequencing was used to study the fungal communities in three fens in the Sanjiang Plain, located at the southern edge of northern peatlands. Peat soil was collected from the three fens which developed during different periods. A total of 463,198 fungal ITS sequences were obtained, and these sequences were classified into at least six phyla, 21 classes, more than 60 orders and over 200 genera. The fungal community structures were distinct in the three sites and were dominated by *Ascomycota* and *Basidiomycota*. However, there were no significant differences between these three fens in any α-diversity index (p > 0.05). Soil age and the carbon (C) accumulation rate, as well as total carbon (TC), total nitrogen (TN), C/N ratio, and bulk density were found to be closely related to the abundance of several dominant fungal taxa. We captured a rich fungal community and confirmed that the dominant taxa were those which were frequently detected in other northern peatlands. Soil age and the C accumulation rate were found to play important roles in shaping the fungal community structure.

## Introduction

Peatlands, worldwide and particularly in the northern (boreal and subarctic) part, are shown to be important participant in global C cycle in the recent past [1]. Despite covering only 6–8% of the terrestrial ecosystems, northern peatlands store 550Pg C [2], accounting for 25–33% of the world’s soil C [3]. The rate of C accumulation is controlled more by changes in annual plant productivity than by decomposition [4]. In particular, the reason for peatlands functioning as a C sink is that more C is fixed by its vegetation than it is lost through the outflow of dissolved organic C and emissions of CO_2_ and CH_4_ [5, 6]. However, there is a growing concern that the ongoing and future global warming will change the C cycling in these ecosystems, making peatlands return the previously captured C to the atmosphere via releasing CO_2_ and/or CH_4_, which would possibly accelerate the pace of global warming [7–10].

Many studies have shown that fungi play a crucial role in the soil ecosystem [11–13]. For example, mycorrhizal fungi contribute to the growth of plants by providing root-available nitrogen and phosphorus [14]. During the initial stage of plant litter decay, saprophagous fungi are more important than bacteria in the degradation of fresh litter into less labile organic matter [15]. The fungal communities, activities, and effects in the peatlands of America, Europe, and Canada have been studied well. Lin et al. [16] found that dominant fungal taxa at bog and fen sites in the peatlands of northwestern Minnesota were Ascomycota, Basidiomycota, and Zygomycota, and the ammonia, dissolved organic carbon (DOC), and dissolved organic nitrogen (DON) concentrations were more important than pH in structuring fungal community structure. The same dominant fungal taxa were detected in the ombrotrophic peatlands of northern England by Liu et al. [17]. They determined that fungal community composition was significantly affected by organic matter (OM), moisture, phosphorus, and ammonium. Sun et al. [18] reported that *Ascomycota* and *Basidiomycota* were the majority groups in the boreal peatlands in Finland, and the dominant tree species and the availability of certain nutrients (Ca, P, and Fe) had a strong effect on the composition of fungal communities. Myers et al. [19] stated that fungal activity became increasingly important and was negatively correlated with pH and total peat Mg concentrations in ombrotrophic peatlands located in southern Canada. Soil age and the C accumulation rate were reportedly to be important factors of fungal community structure in other ecosystems. Several studies found that fungal communities shifted significantly across chronosequences in a forest ecosystem [20–22]. Zhang et al. [23] found that C accumulation promoted macroaggregates formation and reduced the effective diffusion coefficient of oxygen; thus, they identified changes in microhabitats and a shift in microbial community when they investigated the linkage between organic C accumulation and microbial community dynamics in a sandy loam soil.

However, there are few studies on the fungal community in the Sanjiang Plain, leading to the lack of a high-quality curated database for taxonomic assignment. The Sanjiang Plain, located in the temperate climate region, is the largest area of freshwater marshlands in China [24]. These peatlands could be more vulnerable to global warming because they are primarily located at the southern boundary of northern peatlands [1]. Thus, it is imperative that we deepen our understanding of the microorganisms, particularly fungi, in this ecosystem. In this study, high-throughput Illumina sequencing of ITS rRNA genes was used to study the fungal communities in the Sanjiang Plain at the southern edge of northern peatlands. Peat soils were collected from three fens which began development at different time periods. The greater sequencing detail achieved by the high-throughput sequencing allowed the capture of the less abundant and uncultured taxa, thus enabing a more thorough characterization of peatland fungal diversity. The chronological sampling approach further facilitated the analysis of potential linkages of fungal communities with soil age as well as the C accumulation rate.

## Materials and methods

### Study area and sampling description

**Fig 1:**
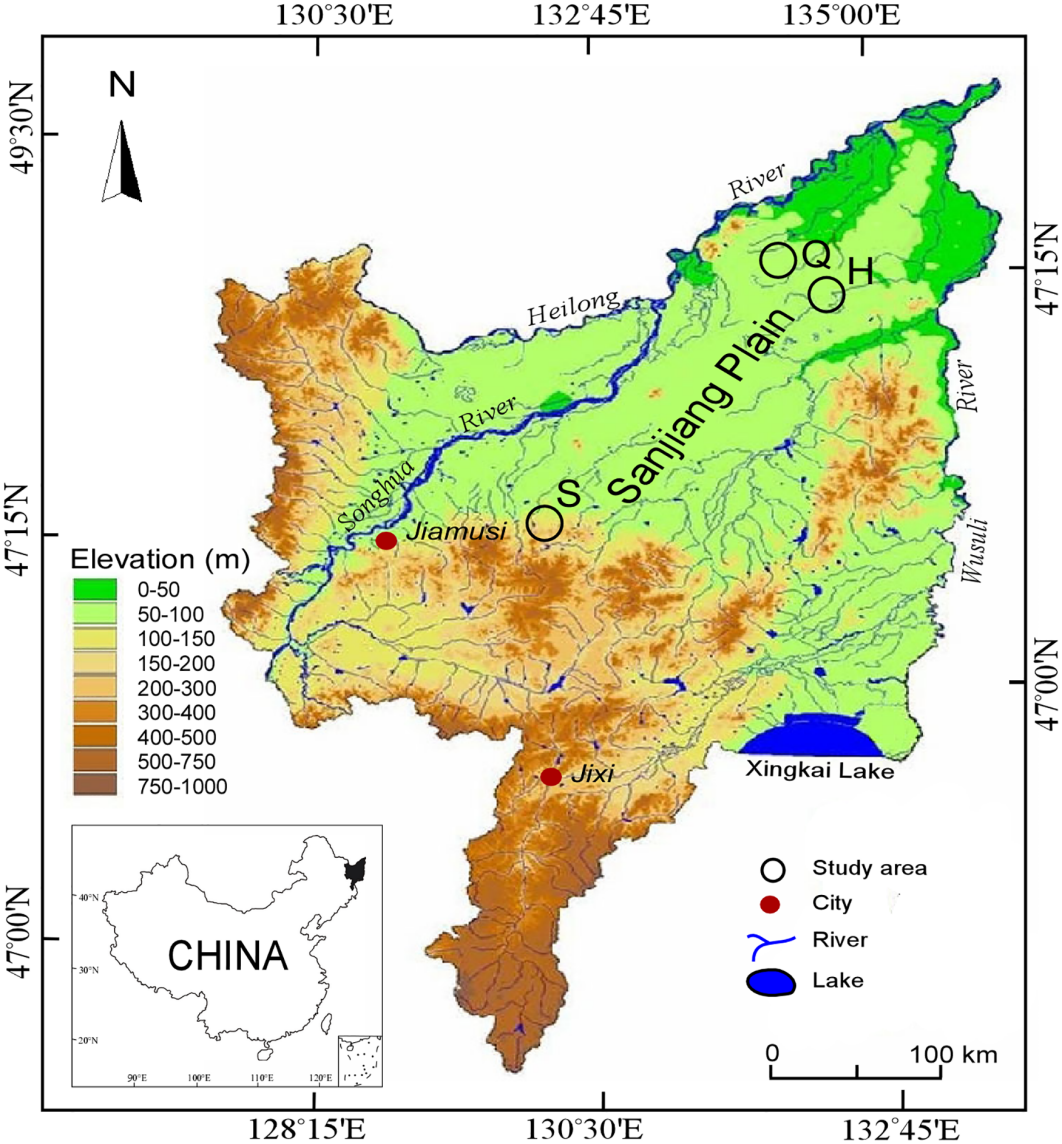
Map of the study region showing locations of the sampling sites in the Sanjiang Plain, which was generated using ArcGIS 10.0 (http://www.esrichina.com.cn/).

Peat cores were sampled from three different minerotrophic fens Shenjiadian (S), Honghe (H), and Qindelie (Q) in the Sanjiang Plain (129°11′-135°05′ E, 43°49′-48°27′ N), China (Fig 1). No specific permits were required for our field studies and our work did not involve endangered or protected species. The three fens started to develop during different time periods during the Holocene [25]. Over 70% of this region is dominated by fresh water wetlands developing in ancient riverbeds and waterlogged depressions, and approximately 30% (or nearly 3.3 × 10^4^ ha) of this region is covered by peatlands, which developed under certain topographic conditions during or before the Holocene [26]. The study area has a temperate humid to sub-humid continental monsoon climate. The mean annual temperature ranges from 1.4°C to 4.3°C, with an average maximum of 22°C in July and an average minimum of -18°C in January. The mean annual precipitation is 500–650 mm, and 80% of rainfall occurs between May and September [27]. The sampled fens receive water inputs from groundwater as well as precipitation and are primarily covered with sedges (*Carex lasiocarpa*). In May 2012, triplicate cores were sampled from each fen using a Russia peat core. The cores were subsampled for chronological, microbiological, and physico-chemical analyses. The soil sample handling for chronological analysis is described in more detail previously [25]. For microbiological and physico-chemical analyses, surface peat soil (depth, 0–30 cm) was homogenized in sterile bags. The peat soils were stored in the dark and kept chilled before they were transferred to a laboratory. Chronological and physico-chemical characterization was conducted right after the samples arrived. Samples for DNA extractions were immediately frozen at -80°C.

### Chronological and physico-chemical characterization

Subsamples with a volume of 3 cm^3^ were used for loss-on-ignition (LOI) with sequential combustion at 500°C to estimate organic matter content [28]. Bulk density was calculated at 1-cm intervals based on the dry weight and volume of each subsample. Ash-free (organic matter) bulk density was calculated from the measurements of the bulk density and organic matter contents. All subsamples for accelerate mass spectrometry (AMS) dating were dated with an accelerator mass spectrometry system at the Institute of Earth Environment, CAS. The AMS ^14^C dates were converted into calendar ages using the program Calib 7.02 based on the INTCAL 13 calibration dataset [29]. The apparent C accumulation rates were calculated using calibrated AMS ^14^C ages, ash-free bulk density measurements, and the C contents of peat organic matter in peatlands (using 52% C in peat organic matter) [30]. Soil pH was measured in a 1:5 soil/water suspension [31]. The total nitrogen (N) in the soil was determined by dichromate oxidation using the continuous flow analytical system (SAN++, SKALAR, Netherlands).

### DNA extraction and sequencing

Genomic DNA was extracted from 0.5 g of peat soil using a FASTDNA^™^ SPIN Kit for soil (MPBio, Santa Ana, CA, USA), according to the manufacturer’s instructions. DNA concentration was measured using a NanoDrop 2000 spectrophotometer (NanoDrop Technologies, Inc., Wilmington, DE, USA). The fungal ITS2 region was amplified using the primer pair ITS3F (GCATCGATGAAGAACGCAGC) and ITS4R (TCCTCCGCTTATTGATATGC) combined with Illumina adapter sequences and barcodes [32]. PCR reactions were performed in a 30-μL mixture containing 3 μL of each primer (2 μM), 10 μL of template DNA (1 ng/μL), 15 uL of Phusion^®^ High-Fidelity PCR Master Mix (BioLabs, Inc., New England, USA), and 2 μL of water. The following thermal program was used for amplification: 95°C for 1 min, followed by 30 cycles of 98°C for 10 s, 50°C for 30 s, 72°Cfor 30 s, and a final extension step at 72°C for 5 min. Each sample was amplified in triplicate, and the PCR products were pooled and purified using the Qiagen Gel Extraction Kit (Qiagen, Hilden, Germany). Metagenomic sequencing libraries were generated using the TruSeq^®^ DNA PCR Free Sample Preparation Kit (Illumina, San Diego, CA, USA), according to the manufacturer’s instructions and pooled at an equimolar ratio. The 250-bp paired-end sequencing was performed on an Illumina HiSeq2000 platform at the Novogene Bioinformatics Technology Ltd.

### Sequence data preprocessing and statistical analysis

Raw sequences were divided into sample libraries via sample-specific barcodes and were truncated after cutting off the barcode and the primer sequence. Forward and reverse reads with at least 10-bp overlaps and less than 5% mismatches were merged using FLASH [33]. Quality filtering on the raw tags was performed according to the QIIME (V1.7.0, http://qiime.org/index.html) quality control process [34], and all sequences shorter than 200 bp with an average quality score lower than 25 in the raw reads were removed. The remaining sequences were subjected to chimera removal using UCHIME Algorithm (http://www.drive5.com/usearch/manual/uchime_algo.html). UPARSE (Version 7.0.1001, http://drive5.com/uparse/) was used to classify the operational taxonomic units (OTUs) at the 97% similarity level [35]. The taxonomic identity was annotated using a BLAST algorithm against sequences within the Unite Database (https://unite.ut.ee/) using the QIIME software [36]. To study the phylogenetic relationship of different OTUs and the differences between the dominant species in different samples (groups), multiple sequence alignments were performed using the MUSCLE software (Version 3.8.31, http://www.drive5.com/muscle/) [35]. Alpha and beta diversity analyses were performed using harmonized data by random subsampling of 43,303 sequences. The alpha diversity indices, including Chao1, Shannon [37], Simpson, ACE, and PieLou equitability [38], were calculated. Beta diversity between microbial communities was evaluated using both Unifrac (weighted and unweighted) and Jaccard distances. Principal coordinate analysis (PCoA) and canonical correspondence analysis (CCA) were performed using the ape and vegan packages in R, respectively. A linear discriminant analysis (LDA) effect size (LEfSe) algorithm was used for high-dimensional biomarker discovery [39] using the non-parametric factorial Kruskal-Wallis (KW) sum-rank test [40] to detect the features with having significant differential abundance with respect to the class of interest; biological consistency was subsequently investigated using with a set of pairwise tests among subclasses using the (unpaired) Wilcoxon rank-sum test [41]. As the final step, LEfSe used LDA to estimate the effect size of each differentially abundant feature and perform dimension reduction, when necessary. Pearson correlation analyses were used to correlate the relationships between the geochemical and microbial parameters of the soil [42]. Differences in soil properties and alpha diversity indices across samples were determined using ANOVA, which was followed by the least significant difference (LSD) test performed in IBM SPSS (version 19.0, Chicago, IL, USA) [43]. The beta-diversity community was compared by permutational MANOVA [44]. All the OTUs were assigned to functional group by FUNGuild [45]. The Illumina sequencing data in the present study has been deposited into NCBI SRA database with the accession number as SRP082472.

## Results

### Physico-chemical and chronological characterization of peat

A total of nine peat cores were retrieved from S, H, and Q fens in the Sanjiang Plain, northeastern China. The AMS dating results indicated that these fens had developed during different periods and had different C accumulation rates. The peat was dated from 637 to 2,085 cal. yr BP (Table 1). Peat cores from Q exhibited the most ancient chronological dates, being dated to 924–2085 cal. yr BP, and S was the youngest fen at 637–862 cal. yr BP. The C accumulation rates of the peat cores ranged from 9.29 to 205.66 g C·m^−2^ yr^−1^ (Table 1).

**Table 1:** Soil properties including the AMS dating result and C accumulation rate of samples from nine peat cores in the Sanjiang Plain.

Sample	Location	Total C (g·kg^-1^)	Total N (g·kg^-1^)	C/N ratio	pH	Bulk density (mg·cm^-3^)	AMS ^14^C age (^14^Cyr BP)	C accumulation rate (g C·m^-2^ yr^-1^)
S1	Shenjiadian1	340.62	21.62	15.75	5.24	0.588	863	101.37
S2	Shenjiadian2	291.15	11.94	24.38	5.38	0.535	820	84.85
S3	Shenjiadian3	316.33	11.62	27.22	5.31	0.462	637	36.98
H1	Honghe1	336.08	15.01	22.39	4.98	0.390	1342	45.39
H2	Honghe2	286.88	19.11	15.01	4.91	0.577	683	70.92
H3	Honghe3	423.85	34.05	12.45	5.44	0.574	764	205.66
Q1	Qindelie1	430.12	16.10	26.72	4.80	0.335	2085	9.29
Q2	Qindelie2	382.54	15.28	25.04	5.14	0.448	924	56.74
Q3	Qindelie3	378.24	15.57	24.30	4.96	0.590	1020	61.72

A summary of soil physico-chemical characteristics was presented in Table 1. Soils were acidic; pH ranged from 4.80 to 5.44. Soil total C and N ranged from 286.88 to 430.12 g kg^−1^ and from 11.62 to 34.05 g kg^−1^, respectively.

### Fungal community diversity

A total of 463,198 sequences targeting the ITS gene were obtained from nine surface (0–30 cm) soil samples using Illumina HiSeq sequencing, ranging from 43,303 to 57,269 reads per sample. After OTU clustering at 97% sequence identity, a total of 989 OTUs were subsequently generated after resampling with 43,303 sequences per sample. Shannon, Simpson, Chao1, ACE index, and PieLou equitability were calculated to estimate microbial richness and evenness (Table 2). However, there were no significant differences between these three fens in all diversity indices (p > 0.05).

**Table 2:** Diversity indices of fungal communities in Shengjiadian (S), Honghe (H), and Qindelie (Q) fens.

Sample name	Sequence read	OTU number	Shannon	Simpson	Chao1	ACE	PieLou equitability
S1	52185	207	1.516	0.381	240.6	245.2	0.197
S2	51787	225	3.561	0.840	235.0	234.8	0.4557
S3	50848	212	3.338	0.832	239.9	240.5	0.432
H1	56985	236	3.076	0.752	251.8	249.5	0.390
H2	50403	192	4.222	0.886	192.8	193.9	0.505
H3	43303	225	3.947	0.857	354.0	254.2	0.487
Q1	54393	232	3.829	0.852	245.6	248.0	0.554
Q2	57269	299	4.558	0.920	334.2	345.1	0.575
Q3	46025	429	5.027	0.916	450.5	451.5	0.557

### Microbial community composition

**Fig 2:**
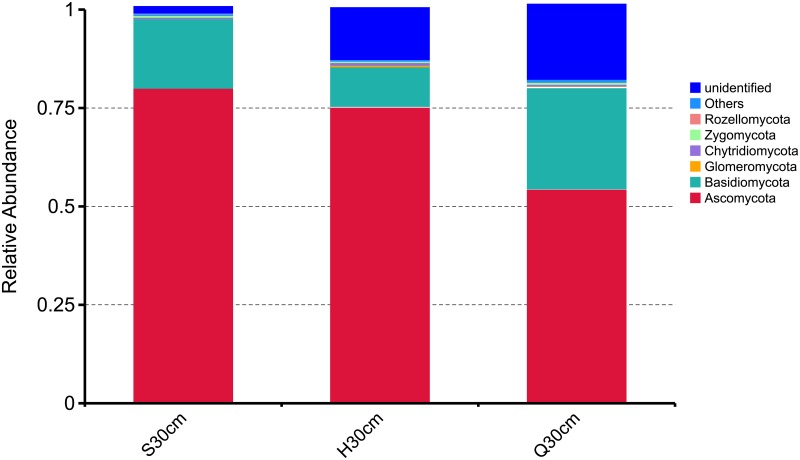
Fungal community variation in the Shengjiadian (S), Honghe (H), and Qindelie (Q) fens. The mean relative abundance of fungi from three cores taken from each site. Each bar height represents the relative abundance, and color represents a particular fungal phylum.

**Fig 3:**
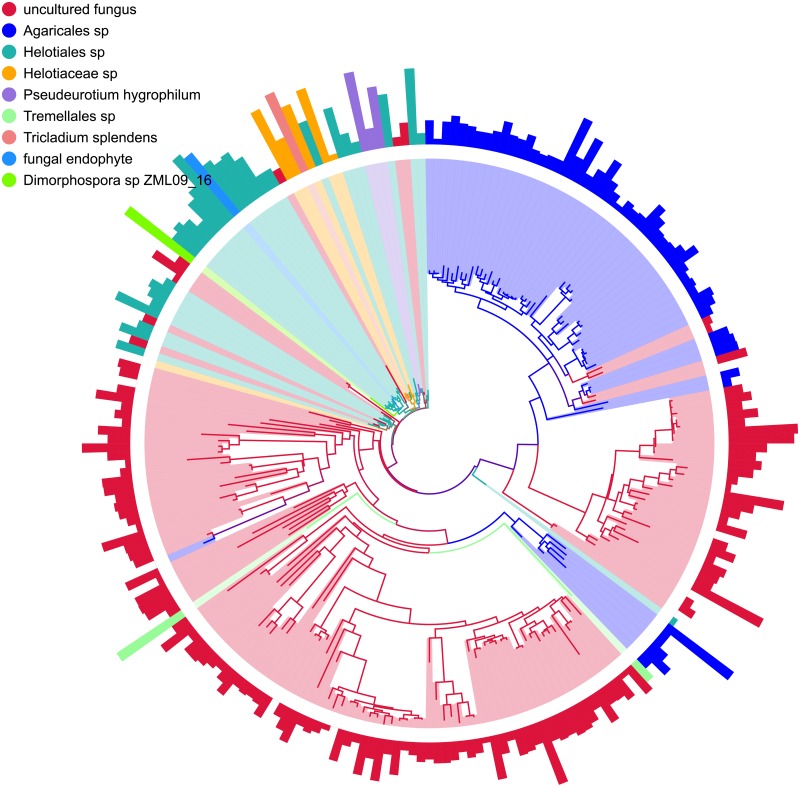
Phylogenetic tree of the OTUs belonging to the top 10 genera. Each color represents a particular OTU. The second and the outermost layer indicate relative abundance and confidence, respectively.

The obtained 463,198 fungal ITS sequences were classified into at least six phyla, 21 classes, more than 60 orders, and over 200 genera, suggesting a rich fungal community in this ecosystem. The dominant fungal phyla across all soil samples were *Ascomycota* and *Basidiomycota*, with relative abundances ranging from 79.75% to 54.07% and 10.04% to 25.38%, respectively (Fig 2). The relative abundances of the minor phyla *Chytridiomycota*, *Zygomycota*, *Glomeromycota*, and *Rozellomycota* were all lower than 1%. In addition, numerous sequences could not be classified into known fungi, with relative abundances varying from 1.65% to 19%. The 10 most abundant fungal OTUs were found to belong to two different phyla (*Ascomycota* and *Basidiomycota*) and three different orders (*Agaricales*, *Helotiales*, and *Tremellales*) (Fig 3).

Only 43.28% of the OTUs were assigned to a functional group by FUNGuild (S1 Table). We detected 14 categories, broadly referred to as guilds; animal pathogens (1.65%), arbuscular mycorrhizal fungi (1.42%), ectomycorrhizal fungi (3.30%), ericoid mycorrhizal fungi (0.35%), lichenized fungi (0.94%), plant pathogens (4.25%), undefined saprotrophs (22.05%), wood saprotrophs (1.18%), endophytes (2.36%), dung saprotrophs (0.83%), root associated biotrophs (0.12%), soil saprotrophs (0.47%), fungal parasites (4.25%), and plant saprotrophs (0.12%). Most of the OTUs were saprotrophs.

**Fig 4:**
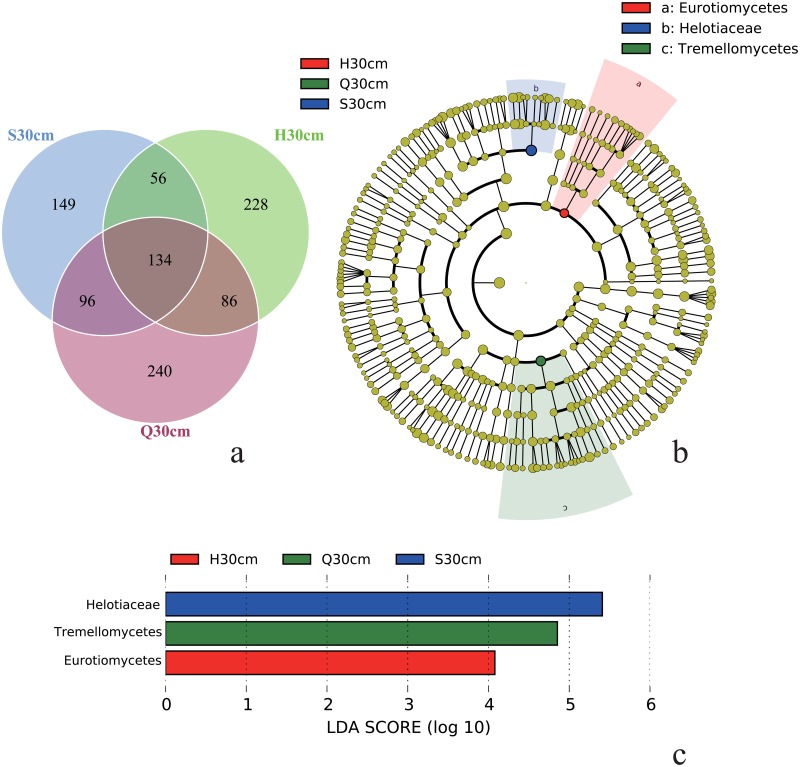
Venn diagram (a) and LEfSe anaylsis (b and c) show the unique and shared OTUs between Shengjiadian (S), Honghe (H), and Qindelie (Q) fens. In the cladogram (b), the circles radiating from the inside out represent fungal taxon from kingdom to family, and the diameter of the circle is proportional to the relative abundance of each taxon. A taxon with significant difference is marked with the same color as the sampling site where the taxon is ranked the highest, and the branch area is correspondingly shaded. A taxon without a significant difference is marked in yellow. Graph (c) is a histogram of the LDA score, and fungal groups that are statistically significant among sites are shown (p < 0.05).

A Venn diagram was used to show a comparison of the similarities and differences between the communities in the three fens (Fig 4a). The Shenjiadian, Honghe, and Qindelie fungal communities had 134 OTUs in common and 149, 228, 240 unique OTUs, respectively. The unique OTUs accounted for 34%, 45% and 43% of the total detected OTUs in Shenjiadian, Honghe, and Qindelie fens. The fungal diversity in these fens was rich, with OTU number, Shannon index and Chao1 index ranging from 192 to 429, 1.516 to 5.027, 192.8 to 450.5, respectively. The fungal community structures were distinct in the three sites (permutational MANOVA, r = 0.375, p = 0.035) and dominated with sequences belonging to *Ascomycota* (Fig 1).

**Fig 5:**
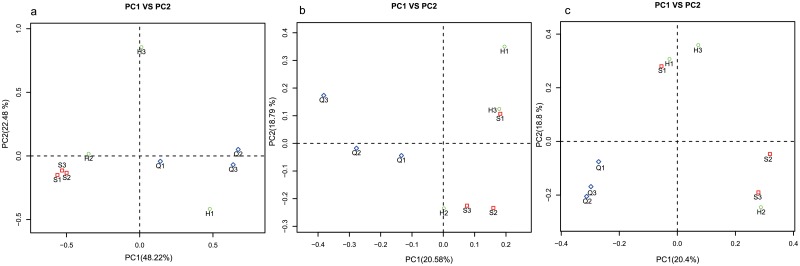
Similarity in fungal community structures indicated by PCoA plots based on weight UniFrac (a), unweight UniFrac (b) and Jaccard (c) distances among sites.

To show the fungal community structures of S, H, and Q fens, PCoA based on both Unifrac and Jaccard distance was performed (Fig 5). The highest relative abundance (80%) of *Ascomycota* sequences was observed in Shenjiadian, the youngest fen. The three sites showed a similar relative abundance of *Zygomycota* (0.35%-0.48%) sequences, while the *Glomeromycota* and *Chytridiomycota* sequences were approximately five-fold and two-fold higher in Honghe than in the other two sites.

The biomarkers explored using the LEfSe analysis were affiliated with *Helotiaceae*, *Tremellomycetes*, and *Eurotiomycetes*, respectively (Fig 4b). According to the Pearson correlation analysis, the relative abundance of *Helotiaceae* was positively correlated with the C accumulation rate (r = 0.706, p = 0.033), and the relative abundance of *Tremellomycetes* was positively correlated with soil age (r = 0.680, p = 0.044). However, the relative abundance of *Eurotiomycetes* was not significantly correlated with any of measured soil parameter (p > 0.05).

### Fungal distribution link to the soil properties

We used CCA to analyze the variation in fungal community structure and the associated soil parameters. Soil parameters showing significant correlation (p < 0.05) with the fungal community were plotted as vectors (Fig 5). On the horizontal axis (CCA1, 19.30% of constrained variability), the most influential constraining variable was C/N ratio (biplot score = 0.82), followed by bulk density (biplot score = -0.36), TN (biplot score = -0.20), C accumulation rate (biplot score = -0.11), soil age (biplot score = 0.07), and TC (biplot score = 0.02). On the vertical axis (CCA2, 18.60% of constrained variability), the most influential constraining variable was soil age (biplot score = 0.61), followed by TC (biplot score = 0.52), C accumulation rate (biplot score = -0.45), bulk density (biplot score = -0.36), C/N ratio (biplot score = 0.29), and TN (biplot score = -0.15).

**Fig 6:**
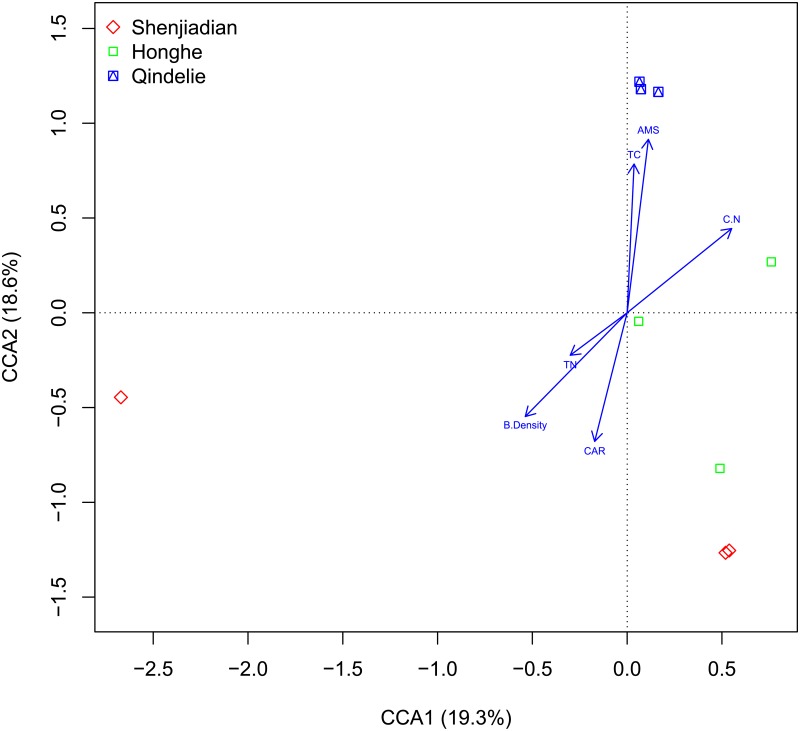
Canonical correspondence analysis (CCA) used to evaluate the effect of soil factors on the fungal community structure. Soil factors indicated in blue text include TC (total carbon), TN (total nitrogen), C/N (C/N ratio), C.A.R. (carbon accumulation rate).

Pearson correlation analyses were used to correlate the relationships between soil properties and the relative abundance of the 10 most abundant fungal taxa at different levels (Fig 6). At the phylum level, the relative abundance of only *Basidiomycota* was significantly (r = 0.708, p = 0.033) correlated with the soil C/N ratio positively. Another dominant phylum exhibited no correlation with measured soil properties. The correlation analysis of the dominant fungal classes and soil properties revealed that the abundance of both *Lecanoromycetes* and *Eurotiomycetes* increased with increasing TN and C accumulation rate. At the order level, we found that the relative abundance of *Sebacinales* was positively correlated with TN (r = 0.890, p = 0.001) and the C accumulation rate (r = 0.847, p = 0.004). *Pleosporales* was negatively correlated with soil bulk density (r = -0.727, p = 0.026) and positively correlated with soil age (r = 0.873, p = 0.002). *Filobasidiales* also exhibited a positive correlation with soil age (r = 0.681, p = 0.043). No significant correlation between soil properties and the relative abundance of fungal taxa was observed at the genus level. Further, among all the measured soil properties, soil pH was the only one which was found to be not correlated with fungal abundance at any level (data not shown).

## Discussion

The fungal taxa observed in the present study primarily belonged to two phyla: *Ascomycota* and *Basidiomycota*, which is consistent with previous studies of peat soils [46–48]. Previous studies reported that *Ascomycota* and *Basidiomycota* were capable of aerobically degrading dissolved organic matter (DOM), including cellulose and polyphenolic compounds [49]. The predominance of *Ascomycota* and *Basidiomycota* at the surface-level peat soil was consistent with this ability. The relative abundance of *Ascomycota* and *Basidiomycota* at the three sites were varied, but the relative abundance of *Zygomycota* among these sites was similar (Fig 2), suggesting that the physiology of *Zygomycota* may distinct from that of *Ascomycota* and *Basidiomycota*. *Zygomycota* can survive over long periods of dormancy by producing thick-walled, resistant spores [12]. Additionally, members of *Zygomycota* are not capable of using cellulose and sucrose degradation products, but instead, they could use C substrates of animal and fungal origin, such as fungal hyphae. For example, an important member of *Zygomycete*, *Mortierella* sp., was observed in all samples. They can degrade chitin, the essential component of fungal hyphae, as efficiently as chitinolytic actinomycetes [50, 51]. We believe that *Zygomycota* may play a role just as important as *Ascomycota* or *Basidiomycota* in the peatlands carbon cycling. Saprotrophs were the dominant functional group. They play an important role in the degradation of fresh litter to less labile organic matter during the initial stages of plant litter decay [15]. Biomarkers revealed by LEfSe were affiliated with *Helotiaceae*, *Eurotiomycetes* and *Tremellomycetes* (Fig 4b). *Eurotiomycetes* are a class of *Ascomycetes* within the subphylum *Pezizomycotina*. One subclass, *Eurotiomycetidae*, includes xerophiles and psychrophiles, which are producers of toxic and useful secondary metabolites, such as fermentation agents used to make food products and enzymes. It also includes the important genetic model *Aspergillus nidulans*. *Chaetothyriomycetidae*, the other subclass, includes the common black yeasts, some of which are pathogens of humans and animals, as well as some primarily lichen-forming groups newly found to be phylogenetically associated with this group [52]. *Helotiaceae* is a small group of fungicolous, lichenicolous, and discomycetes members [53]. *Tremellomycetes*, a basal group in the *Agaricomycotina*, comprises mostly dimorphic taxa and species that form hyphae and/or complex fruiting bodies [54]. We found that the relative abundance of *Tremellomycetes* and *Helotiaceae* was positively correlated with soil age (r = 0.680, p = 0.044) and the C accumulation rate (r = 0.706, p = 0.033), respectively. Peat samples collected from Shenjiadian and Qindelie had relatively higher C accumulation rate and were older than those collected from Honghe (Table 1). However, the relative abundance of *Eurotiomycetes* was not significantly correlated with any of the measured soil parameters (p > 0.05). Vegetation can affect the fungal community structures and may also be an important factor associated with the biomarker. Although the S, H and Q fens were primarily covered with sedges (*Carex lasiocarpa*), other vegetation and their proportion, such as the amount of shrubs and tree likely differ. Faster C accumulation and older age likely mean that the surface of the Shenjiadan and Qindelie fens were drier (with lower water tables) and that they had a greater percentage of woody species, as a part of the minority vegetation. Further, *Helotiaceae* and *Tremellomycetes* are dominate by terricolous or lichen-forming species, such as Gelatinopsis [55] and Hymenoscyphus [56], two of the largest genera of *Helotiaceae*, and *Tremella* and *Syzygospora* belonging to *Tremellomycete* [57]. The functional roles of these biomarkers in the three fens still remain to be elucidated.

In this study, fungal communities is correlated with soil physico-chemical properties. Both CCA and Pearson correlation analysis indicated that pH is less important as an environmental force in shaping the fungal community structure. This could be attributed to the ability of fungi to tolerate a wide range of pH and the fact that their optimal extracellular enzyme activity is at low pH [58]. This study of only three acidic sites does not cover a broad enough pH range to detect this relationship. Our results also suggested that TC, TN, and C/N ratio were important in structuring fungal distribution, which was consistent with other studies, not only in peatlands [12], but also in farmlands [17, 59] and forests [18]. All of these soil parameters were related to nutrient availability, which may have obvious implications for fungi growth. Furthermore, soil bulk density was identified as a driving force for fungal distribution in this study. Soil bulk density is inversely related to porosity [60] as the pores serve as pathways for water and oxygen, which are important growth factors of fungi [61]. We also found that the fungal community was significantly correlated with soil age and C accumulation in peatlands. The peat cores initiated from different ages, being affected by climatic conditions (e.g., temperature and precipitation), underwent different initiation and decomposition process, which resulted in the respective soil properties [2] We speculate that the effect of soil age on fungal community is due to its correlation with soil properties. Temperature, oxygen exposure time, and vegetation have been proposed as important controls on the C accumulation rate of peatlands and could yield a different C fraction, which may be used as potential substrate or cause an inhibitory affect on microorganism growth [2]. Fungi, as an important participant of C cycling, could change the C accumulation rate of peatlands. The correlation between C accumulation and microbial community has been previously investigated. Mackay et al. found the positive correlations between total organic C accumulation and microbial indicators (total PLFA, fungal PLFA, bacterial PLFA and activities of decomposition enzymes) when they surveyed a chronosequence (0–23 years) of reforesting riparian pastures [62]. Kaƚucka and Jagodziński [63] reported that ectomycorrhizal fungi were either indirectly involved in the C accumulation through their influence on tree biomass production and organic matter decomposition in boreal and temperate forests or directly involved through C storage by the mycelium. A mechanistic understanding of the role of important fungal taxa in peatland C cycling requires further field experiments and ecophysiological studies in the laboratory. However, several studies demonstrated that fungal community distribution pattern is mainly affected by pH, peatland vegetation, DOC, or DON [6, 12, 61]. In addition, hydrology and the hydroperiod dictates the above-ground vegetation, and this in turn is both influenced by and influences fungal and bacterial communities [5]. The peatland fungal community distribution pattern remains to be sorted out as the technology and methodology are developing and each wetland is unique along a hydrological spectrum.

## Conclusions

In conclusion, the composition and diversity of fungal communities in three different minerotrophic fens distributed in the Sanjiang Plain, the southern edge of northern peatlands in China, were investigated by high-throughput Illumina sequencing. We captured a rich fungal community and confirmed that the dominating taxa have also been frequently detected in other northern peatland ecosystems. The fungal community structures were distinct in the three sites, however, there was no significant differences in α-diversity (p > 0.05). Unlike pH, TC, TN, C/N ratio, and bulk density are determined to be important environmental parameters shaping fungal community structure. Additionally, we found that the distribution patterns of several abundant fungal taxa were closely related to the soil age and C accumulation rate. However, because of limited sampling sites in the research, these results should be considered preliminary. Data presented here may serve useful for future researchers in this field. Extensive sampling of a larger range of peatland sites is required to further this research, and may reveal a relationship between the fungal community and pH, which was not detected in this study.

## Supporting information

S1 Fig The relationship between the relative abundances of abundant fungal phyla (A), fungal classes (B), fungal orders (C), fungal genera (D) and soil properties. (JPG)

S1 Table Annotation results assigned by FUNGuild. (XLSX)
